# Predictive factors for survival following stereotactic body radiotherapy for hepatocellular carcinoma with portal vein tumour thrombosis and construction of a nomogram

**DOI:** 10.1186/s12885-021-08469-1

**Published:** 2021-06-15

**Authors:** Xiaojie Li, Zhimin Ye, Sheng Lin, Haowen Pang

**Affiliations:** 1 Department of Oncology, The Affiliated Hospital of Southwest Medical University, Luzhou, 646000 Sichuan China; 2 Department of Radiation Oncology, Cancer Hospital of The University of Chinese Academy of Sciences (Zhejiang Cancer Hospital), Hangzhou, 310022 China

**Keywords:** Hepatocellular carcinoma, Portal vein tumour thrombosis, Stereotactic body radiotherapy, Overall survival, Nomogram

## Abstract

**Background:**

We evaluated the treatment response and predictive factors for overall survival (OS) in patients with hepatocellular carcinoma (HCC) and portal vein tumour thrombosis (PVTT), who underwent stereotactic body radiotherapy (SBRT). Additionally, we developed and validated a personalised prediction model for patient survival.

**Methods:**

Clinical information was retrospectively collected for 80 patients with HCC and PVTT, who were treated with SBRT at the Cancer Hospital of the University of Chinese Academy of Sciences (Zhejiang Cancer Hospital) between December 2015 and June 2019. A multivariate Cox proportional hazard regression model was used to identify the independent predictive factors for survival. Clinical factors were subsequently presented in a nomogram. The area under the receiver operating characteristic curve (AUC) and decision curve analysis (DCA) were used to evaluate the accuracy of the model and the net clinical benefit.

**Results:**

All patients completed the planned radiotherapy treatment, and the median follow-up duration was 10 months (range, 1–35.3 months). The median survival duration was 11.5 months, with 3-, 6-, and 12-month survival rates of 92.5, 74.5, and 47.5%, respectively. The multivariable Cox regression model indicated that the following were significant independent predictors of OS: clinical T stage (*p* = 0.001, hazard ratio [HR] = 3.085, 95% confidence interval [CI]: 1.514–6.286), cirrhosis (*p* = 0.014, HR = 2.988, 95% CI: 1.246–7.168), age (*p* = 0.005, HR = 1.043, 95% CI: 1.013–1.075), alpha-fetoprotein level (*p* = 0.022, HR = 1.000, 95% CI: 1.000–1.000), and haemoglobin level (*p* = 0.008, HR = 0.979, 95% CI: 0.963–0.994). A nomogram based on five independent risk factors and DCA demonstrated a favourable predictive accuracy of patient survival (AUC = 0.74, 95% CI: 0.63–0.85) and the clinical usefulness of the model.

**Conclusions:**

SBRT is an effective treatment for patients with HCC with PVTT. Notably, clinical T stage, presence of cirrhosis, age, alpha-fetoprotein levels, and haemoglobin levels are independent prognostic factors for survival. The presented nomogram can be used to predict the survival of patients with HCC and PVTT, who underwent SBRT.

## Background

Hepatocellular carcinoma (HCC) is one of the most common malignant tumours, with a high degree of malignancy and a low survival rate [1]. It is estimated to be the fourth most common cause of cancer-related deaths globally [2]. Macrovascular invasion, such as portal vein tumour thrombosis (PVTT) or inferior vena cava tumour thrombosis (IVCTT), is a common complication of advanced hepatocellular carcinoma (HCC), with an incidence rate of 10–40% at the time of initial diagnosis [3, 4]. Although the survival rate after comprehensive treatment for HCC has improved in recent years, patients with both HCC and PVTT still have a poor prognosis. Their median survival duration is 2.7–4 months in the absence of treatment [5, 6]. PVTT formation is often accompanied by portal hypertension, tumour spread, and a deterioration of liver function, partially limiting the applicability of surgical resection and transarterial chemoembolisation (TACE) [7, 8].

Multiple clinical studies have demonstrated that radiotherapy, including three-dimensional conformal radiotherapy (3DCRT), is an effective treatment for PVTT/IVCTT; 3DCRT is the most widely used modality [9–12]. A randomised, open-label, multicentre, controlled study demonstrated that neoadjuvant 3DCRT provided significantly better postoperative survival rates than surgery alone in patients with resectable HCC and PVTT [13]. Stereotactic body radiotherapy (SBRT) is a new radiotherapy technology that can be used to administer precision radiotherapy to a target tumour area with a concentrated target dose and less damage to the surrounding normal tissues, effectively reducing the incidence of adverse reactions while improving the therapeutic effect [14, 15]. Currently, SBRT has been used in the treatment of HCC with PVTT [16, 17]. To shrink the tumour thrombus and maintain sufficient portal venous flow, SBRT is recommended for patients with unresectable HCC having PVTT and for those with contraindications for TACE [18, 19]. Despite the existence of clinical reports on the efficacy and safety of SBRT for HCC with PVTT, to the best of our knowledge, there is no nomogram for predicting survival of patients with HCC and PVTT after undergoing SBRT. Therefore, to further understand the efficacy and prognosis of HCC cases with PVTT after SBRT, this study aimed to evaluate the treatment response and risk factors for survival among 80 patients with HCC and PVTT, who were treated with SBRT. Additionally, this study sought to develop and validate a nomogram based on clinical characteristics to individually predict the survival of patients.

## Methods

### Patient selection and SBRT

We retrospectively reviewed the records of 80 patients with advanced HCC complicated by PVTT, who received SBRT between December 2015 and June 2019 at the Cancer Hospital of the University of Chinese Academy of Sciences (Zhejiang Cancer Hospital). The inclusion criteria were as follows: (1) patients aged between 25 and 75 years who were diagnosed with HCC complicated through PVTT by histopathological or radiological assessment; (2) patients who were ineligible for surgery or with tumours medically unsuitable for resection; (3) an Eastern Cooperative Oncology Group (ECOG) performance status score of 0–2; (4) patients with Child-Pugh class A or B liver function; (5) patients without a history of liver radiotherapy; and (6) patients with a liver volume above 700 cc outside of the planning target volume (PTV). All patients were diagnosed as having PVTT by contrast-enhanced computed tomography (CT), presenting with portal vein lumen thickening and intravascular low-density filling defect. Cheng’s classification was used in this study, which comprises four types based on the extent of PVTT invasion on the portal vein: type I, involvement of the segmental or sectoral branches of the portal vein or above; type II, involvement of the right- or left-side branch of the portal vein; type III, involvement of the main trunk of the portal vein; type IV, thrombus extends to the superior mesenteric vein [13]. All patients provided written informed consent to receive treatment, and the retrospective study was approved by the local ethics committee.

Tumour staging of all patients was based on the 8th edition of the American Joint Committee on Cancer tumour-node-metastasis (TNM) staging criteria [20]. The diagnosis of cirrhosis is primarily confirmed by imaging, including colour ultrasound and CT, or by hipathology. Before SBRT treatment, 37 (46.25%), three (3.75%), and 24 (30.00%) patients had received TACE, radiofrequency ablation (RFA), and combined TACE with RFA, respectively. Oral sorafenib (400 mg, twice a day) was administered to a total of 24 patients (30.00%), before 1 month or less, and the treatment was continued until the patients appeared not to be clinically benefitted or experienced intolerable toxic side effects.

Patients were immobilised with vacuum bags in the supine position with their arms raised above their heads during simulation and treatment. Contrast-enhanced four-dimensional computed tomography (4DCT) was performed in most of the patients with 2.5-mm slice thickness during quiet breathing. Gross tumour volume (GTV) was defined as PVTT. When the primary liver lesion was small (< 5 cm) and adjacent to the PVTT, both were contoured as the GTV. Delineation was performed in each phase on the 4DCT referring to the contrast-enhanced CT and magnetic resonance imaging (MRI) scans. The internal target volume (ITV) was defined as the combined volume of GTVs in the multiple 4DCT phases. For the PTV, individualised margins of 3–5 mm were applied around the ITV to account for inter-fractional motion variability and daily setup errors. The organs at risk included the liver, stomach, duodenum, small intestine, colon, kidneys, and spinal cord. The normal liver volume was defined as the total volume minus the PTV. The mean normal doses of the liver and the bilateral kidney were ≤ 15 and 12 Gy, respectively. The maximal permitted dose to 1 cc (D1cc) was limited to 31 Gy for the stomach, duodenum, small intestine, and colon. The maximum dose to the spinal cord was 27 Gy.

Coplanar fixed-field intensity-modulated radiation therapy plans were devised using Eclipse TPS (Varian Medical Systems, Palo Alto, CA) or Raystation TPS (RaySearch Laboratories AB, Stockholm, Sweden). A total dose of 25–50 Gy in five fractions over 5–7 days was prescribed for the PTV [21]. Photon beams of 6 MV were delivered using a Varian Trilogy linear accelerator (Varian Medical Systems) or Elekta Synergy linear accelerator (Elekta Oncology Systems, Crawley, UK). Cone beam CT scans were acquired and registered for the planning CT prior to every treatment.

### Follow-up and evaluation

The response of PVTT to SBRT was evaluated using contrast-enhanced CT at 1 and 3 months after SBRT, and every 3 months thereafter. The last follow-up visit was conducted on June 1, 2020; the overall survival (OS) was calculated from the start of SBRT to the date of death or that of the last follow-up visit. Follow-up visits involved a clinical evaluation and diagnostic imaging test (CT, MRI, or positron emission tomography). The modified Response Evaluation Criteria in Solid Tumours were used to evaluate the tumour response [22]. All CT and MRI scans were acquired by two experienced radiologists. In accordance with the Common Terminology Criteria for Adverse Events (CTCAE; version 3.0), toxicities of patients were assessed weekly during radiotherapy, at the 1st month after SBRT, once a month for the following 2 months, and then, once every 3 months.

### Statistical analyses

All analyses were performed using R software, version 3.6.3 (R Foundation for Statistical Computing, Vienna, Austria). Survival curves were calculated using the Kaplan–Meier method. Univariate analyses were performed, and multivariate Cox proportional hazard regression models were used to identify the predictive factors of survival. Packages ‘rms’ and ‘Hmisc’ of R language were used to develop and verify the prediction model. The area under the receiver operating characteristic curve (AUC) and the decision curve analysis (DCA) were used to evaluate the accuracy of the model and net clinical benefits. Values of *p* < 0.05 were considered statistically significant.

### Development and validation of an individualised prediction model

To provide a quantitative tool for clinicians to individually predict the survival of patients, we developed a nomogram based on five independent risk factors. The AUC was used to evaluate the nomogram, and calibration curves were plotted.

### Clinical usefulness

DCA was performed to determine the clinical usefulness of the developed nomogram by quantifying the net benefits at different threshold probabilities.

## Results

In total, 80 patients with both HCC and PVTT, treated with SBRT were included in this study. The median age was 54 (range, 25–75) years, and most of the patients were male (83.75%). Prior to SBRT, 61 (76.25%) patients underwent TACE, and 62 (77.50%) had cirrhosis. In addition, 48 (60.00%) patients had tumour thrombosis involving the first-order portal vein branches (Type II). Tumour thrombosis invading the main trunk (Type III) was found in 30 (37.50%) patients, and only two (2.50%) patients had tumour thrombosis that invaded the superior mesenteric vein or the inferior vena cava (Type IV). Furthermore, most patients (82.5%) had underlying viral hepatitis caused by hepatitis B virus (65 patients, 81.25%); only one patient had underlying viral hepatitis caused by hepatitis C virus. In the follow-up period after SBRT, 35 (43.75%) patients underwent TACE, and seven received RFA after TACE. Five patients underwent liver surgery. The other patient characteristics are presented in Table 1.

**Table 1:** Patient characteristics

Characteristics	n (%)
Age, years	
≥ 50	53 (66.25)
< 50	27 (33.75)
Sex	
Male	67 (83.75)
Female	13 (16.25)
ECOG PS	
0	61 (76.25)
1	18 (22.50)
2	1 (1.25)
T stage	
T2	7 (8.75)
T3	65 (81.25)
T4	8 (10.00)
N stage	
N0	58 (72.50)
N1	22 (27.50)
M stage	
M0	66 (82.50)
M1	14 (17.50)
Underlying hepatitis	
Hepatitis B	65 (81.25)
Hepatitis C	1 (1.25)
Negative	14 (17.50)
Cirrhosis	
Yes	62 (77.50)
No	18 (22.5)
Previous treatment	
TACE	37 (46.25)
RFA	3 (3.75)
TACE+RFA	24 (30.00)
None	16 (20.00)
Child-Pugh classification	
A	55 (68.75)
B	25 (31.25)
Radiation dose, Gy	
< 36	30 (37.50)
≥ 36	50 (62.50)
Types of PVTT	
II	48 (60.00)
III	30 (37.50)
IV	2 (2.50)
Combined with sorafenib	
Yes	24 (30.00)
No	56 (70.00)
Additional treatment after SBRT	
Yes	47 (58.75)
No	33 (41.25)
Tumour maximum diameter, cm	
< 5 cm	18 (22.50)
≥ 5 cm	62 (77.50)
AFP, ng/L	
< 20	18 (22.50)
20–400	20 (25.00)
> 400	42 (52.50)
PLT, × 10^9^/L	
> 100	45 (56.25)
≤ 100	35 (43.75)
HGB, g/L	
> 120	48 (60.00)
≤ 120	32 (40.00)
TBIL, gmol/L	
> 20	37 (46.25)
≤ 20	43 (53.75)
ALB, g/L	
> 35	50 (62.50)
≤ 35	30 (37.50)
ALT, U/L	
> 50	27 (33.75)
≤ 50	53 (66.25)
AST, U/L	
> 50	51 (63.75)
≤ 50	29 (36.25)

Abbreviations: PVTT, portal vein tumour thrombus; ECOG PS, Eastern Cooperative Oncology Group performance status; TACE, transarterial chemoembolisation; RFA, radiofrequency ablation; AFP, alpha-fetoprotein; PLT, platelet; HGB, haemoglobin; TBIL, total bilirubin; ALB, albumin; ALT, alanine aminotransferase; AST, aspartate aminotransferase

### Treatment response

After completion of SBRT, PVTT-related therapeutic responses were observed in 72 patients. A complete response (CR) was observed in eight (11.11%) patients and a partial response (PR) was observed in 49 (68.06%) patients; stable disease (SD) was observed in four (5.56%) patients. The remaining 11 patients (15.28%) showed progressive disease. The response rate (CR + PR) was 79.17%. Local control (including CR, PR, and SD) was achieved in 84.72% of the treated lesions.

### Follow-up period and survival

**Fig. 1:**
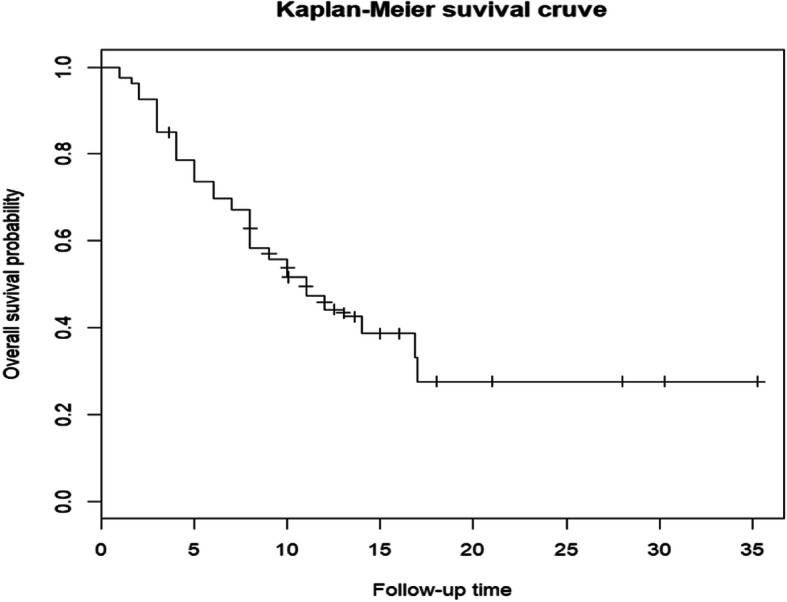
Kaplan–Meier curve of overall survival of all patients

The median follow-up duration was 10 (range, 1–35.3) months. Thirty-two (40%) patients were still alive at the last follow-up examination. The median survival duration was 11.5 months, with 3-, 6-, and 12-month survival rates of 92.5, 74.5, and 47.5%, respectively (Fig. 1).

### Toxicity

No radiation-induced liver disease or grade 4/5 acute treatment-related toxicity was observed within 3 months after SBRT treatment. The most common acute adverse effects were gastrointestinal reactions, such as nausea, vomiting, anorexia, and abdominal distension. Grade 1 or 2 transaminase elevation and grade 3 bilirubin elevation were observed in 10 (12.5%) and seven (8.75%) patients, respectively. Grade 3 acute bone marrow suppression was found in four cases. No other serious toxicities were reported during the follow-up period.

### Prognostic analysis

We used Cox regression hazard models to perform univariate analyses for all patients. The results demonstrated that sex, age, ECOG performance status score, presence of cirrhosis, clinical T stage, Child-Pugh class, alpha-fetoprotein (AFP) levels, albumin (ALB) levels, and haemoglobin (HGB) levels were significantly related to the OS rate. Other factors, such as the clinical N stage, clinical M stage, hepatitis, pre-SBRT treatment, radiotherapy dose, types of PVTT, combined sorafenib treatment, additional treatment after SBRT, tumour maximum diameter, platelet (PLT) count, total bilirubin (TBIL) levels, alanine aminotransferase (ALT) levels, and aspartate aminotransferase (AST) levels, had no statistical significance on the OS rate. The results of univariate analyses for the factors associated with OS are provided in Table 2. Next, a multivariate Cox proportional hazard regression model showed that clinical T stage (*p* = 0.001, hazard ratio [HR] = 3.085, 95% confidence interval [CI]: 1.514–6.286), cirrhosis (*p* = 0.014, HR = 2.988, 95% CI: 1.246–7.168), age (*p* = 0.005, HR = 1.043, 95% CI: 1.013–1.075), AFP levels (*p* = 0.022, HR = 1.000, 95% CI: 1.000–1.000), and HGB levels (*p* = 0.008, HR = 0.979, 95% CI: 0.963–0.994) were significant independent predictors of OS (Table 3).

**Table 2:** Univariate analyses of baseline characteristics

Covariate	HR (95% CI)	*p*-value
Age	1.029 (1.001–1.058)	0.04272
Sex	2.018 (1.027–3.966)	0.04175
ECOG PS	1.924 (1.068–3.465)	0.02932
T stage	1.81 (1.026–3.193)	0.04041
N stage	1.111 (0.595–2.072)	0.7419
M stage	1.237 (0.599–2.557)	0.5651
Underlying Hepatitis	1.062 (0.512–2.202)	0.8725
Cirrhosis	2.25 (1.001–5.059)	0.04976
Previous treatment	0.555 (0.288–1.071)	0.07916
Child-Pugh classification	2.558 (1.454–4.501)	0.001122
Radiation dose	0.973 (0.921–1.028)	0.3274
Types of PVTT	1.151 (0.936–1.416)	0.1822
Combined with sorafenib	0.99 (0.968–1.013)	0.4064
Additional treatment after SBRT	0.999 (0.998–1)	0.1453
Tumour maximum diameter	0.971 (0.82–1.151)	0.7344
AFP	1 (1–1)	0.03041
PLT	1.001 (0.996–1.005)	0.8191
HGB	0.978 (0.962–0.995)	0.009121
TBIL	1.008 (0.997–1.019)	0.1405
ALB	0.906 (0.849–0.968)	0.003482
ALT	0.997 (0.993–1.002)	0.2489
AST	1 (0.998–1.001)	0.5813

Abbreviations: HR, hazard ratio; CI, confidence interval; PVTT, portal vein tumour thrombus; ECOG PS, Eastern Cooperative Oncology Group performance status; TACE, transarterial chemoembolisation; AFP, alpha-fetoprotein; PLT, platelet; HGB, haemoglobin; TBIL, total bilirubin; ALB, albumin; ALT, alanine aminotransferase; AST, aspartate aminotransferase

**Table 3:** Multivariate analyses of baseline characteristics

Covariate	Hazard ratio (95% confidence interval)	p-value
T stage	3.085 (1.514–6.286)	0.001925
Cirrhosis	2.988 (1.246–7.168)	0.0141971
AFP	1 (1–1)	0.0229561
HGB	0.979 (0.963–0.994)	0.0081922
Age	1.043 (1.013–01.075)	0.0051956

AFP, Alpha-fetoprotein; HGB, Haemoglobin

### Development and validation of an individualised prediction model

**Fig. 2:**
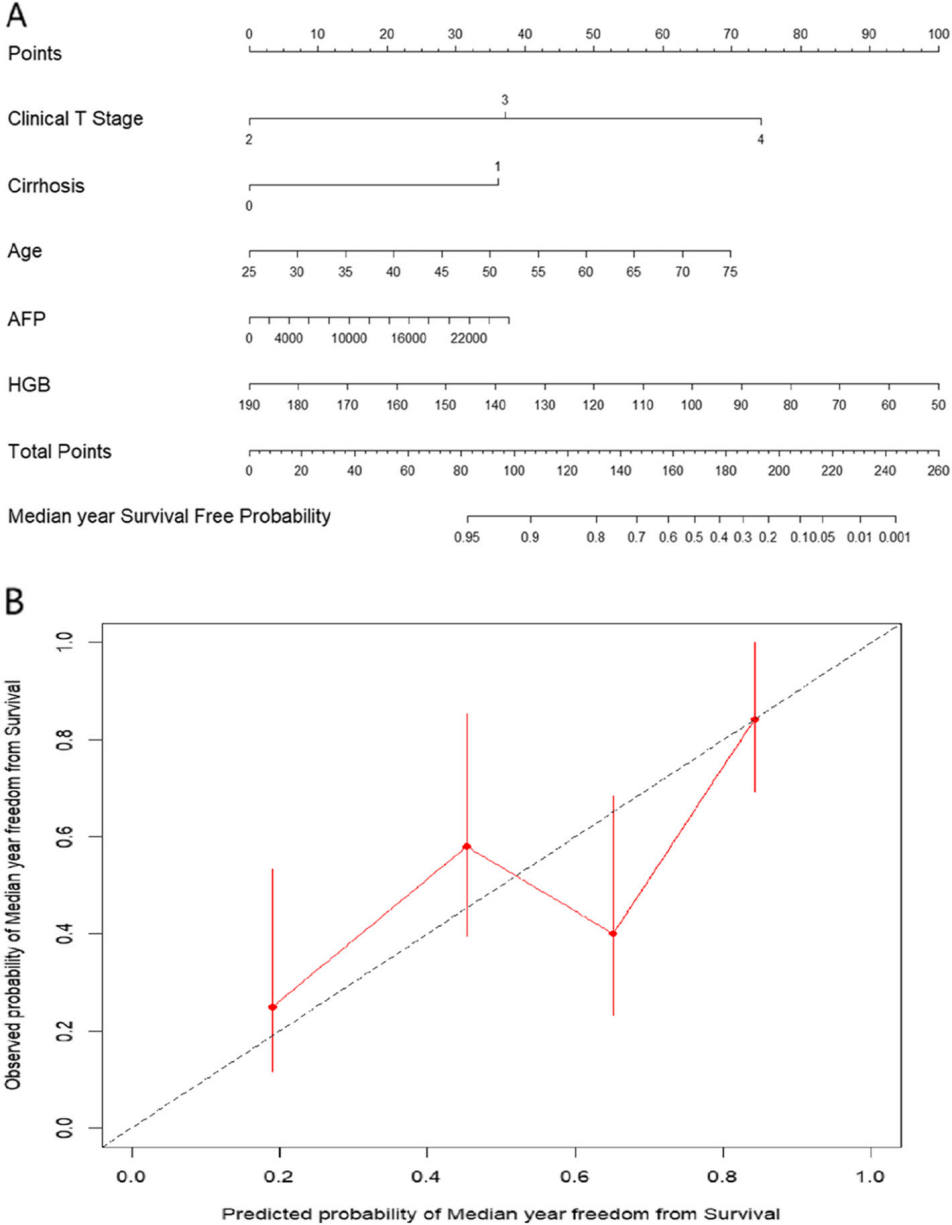
(a) Nomogram established for median survival prediction based on five indicators. (b) Nomogram calibration curves

**Fig. 3:**
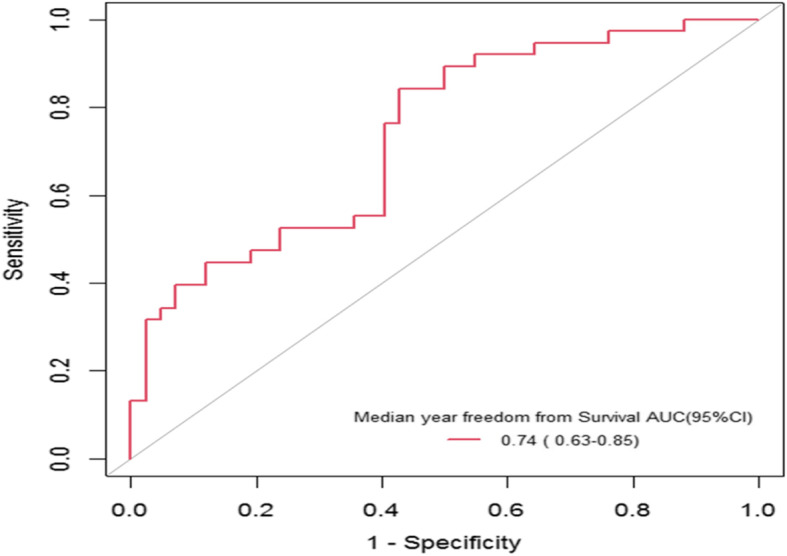
Receiving operating characteristics curve of median survival

The nomogram, based on five independent risk factors, and the calibration curves are presented in Fig. 2. The calibration curve for the probability of median survival showed a moderate level of consistency between the predictions and observations. The AUC for the nomogram was 0.74 (95% CI: 0.63–0.85) (Fig. 3), and the diagnostic value was favourable, conferring a certain degree of significance in the prediction of individual survival.

### Clinical usefulness

**Fig. 4:**
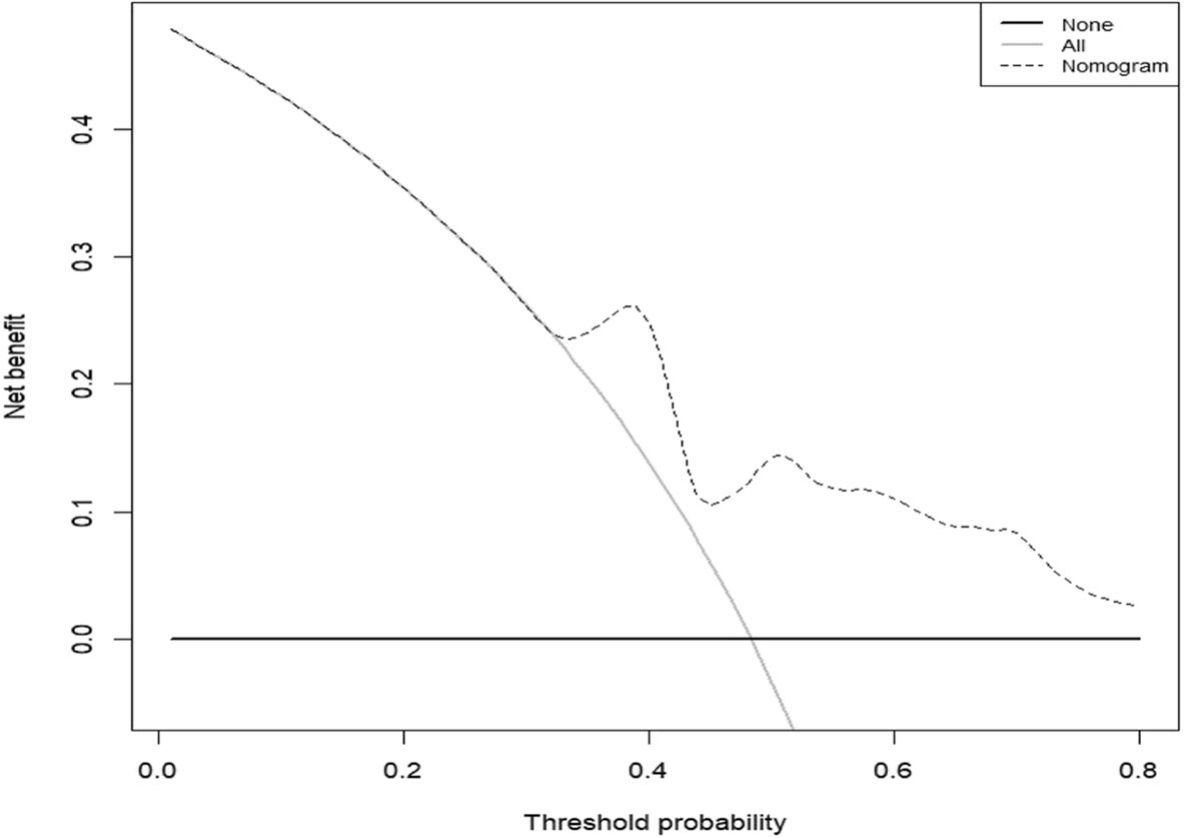
Decision curve analysis for the nomogram based on clinical characteristics. The broken line represents the nomogram. The thick line represents all negative samples. These participants received no interventions. The fine line represents all positive samples, and these participants received interventions

The DCA result for the nomogram is presented in Fig. 4. The decision curve demonstrated that if the threshold probability of a patient or physician was 34%, using the developed nomogram to predict the median survival was more beneficial than using the treat-all-patients or treat-none schemes. For instance, when the personal threshold probability of a patient was 40% (especially, the patient would choose to receive treatment when the probability of cancer was > 40%), the net benefit would be 0.25. The developed nomogram was more beneficial than the treat-all scheme or the treat-none scheme in determining whether to receive treatment.

## Discussion

In patients with HCC and PVTT, the treatment options are limited, and the optimal treatment remains controversial [23]. TACE is often used for HCC lesions that cannot be surgically resected. Although the use of TACE alone has a certain curative effect in patients with HCC and PVTT, the effect is far smaller than expected. In patients with poor liver function and complete portal vein embolisation without collateral circulation, TACE is usually contraindicated. This is mainly because the blood supply to the liver involves the hepatic artery and the portal vein; when the hepatic artery is re-embolised after portal vein obstruction, the liver receives no blood supply, leading to liver tissue necrosis and liver failure.

The Barcelona Clinic Liver Cancer (BCLC) Staging System is the most widely adopted HCC management guideline; according to this system, HCC with PVTT is considered as a BCLC stage-C disease [8]. In addition, the guidelines recommend sorafenib administration as the standard treatment for advanced HCC; however, the survival benefits observed after treatment with sorafenib remain limited for patients with HCC and PVTT. Therefore, it is essential to explore alternative or combination therapies to improve the prognosis of patients with HCC and PVTT [23].

According to clinical radiobiology, HCC tissue is an early reactive tissue (α/β > 10 Gy), and HCC tumours are radiosensitive [24]. However, the radiation dose tolerated by liver cells is lower than the radical dose delivered to liver cancer cells; furthermore, conventional radiotherapy is not compatible with a high dose of radiation because of the large irradiation volume and damage to liver function, thus resulting in serious adverse reactions and poor therapeutic effects [25]. Although in recent years, many studies have reported that radiotherapy is effective for PVTT, its efficacy is also limited by the maximum tolerated dose of hepatocytes and severe adverse reactions [10, 12]. In recent years, with the development of stereotactic radiotherapy and its wide application in clinical practice, the dilemma linked to routine radiotherapy for patients with liver cancer has been modified, thereby providing a new approach for the treatment of PVTT.

As an accurate external radiotherapy technology, stereotactic radiotherapy is applied to the tumour lesion area for aggregated radiotherapy with a steep dose curve, significantly improving the curative effect and reducing the incidence of radiation reactions and the degree of the normal tissue damage [26]. Dang et al. [26] reported the curative effect of SBRT on hepatic hilar carcinoma and confirmed that SBRT is a safe and reliable treatment method for primary liver cancer. Moreover, they highlighted its efficiency in improving patient survival. In the study by Zeng et al. [27], 121 patients with a cancer suppository who received radiotherapy had a median survival duration of 8.9 months and a 1-year survival rate of 34.4%; in contrast, the median survival duration in the non-radiotherapy control group was 4 months, and the 1-year survival rate was 11.4% in the same period. Our results further confirmed the efficacy of radiotherapy in patients with HCC and PVTT. A study by Xi et al. [17] reported a median survival of 13 months and a 1-year OS rate of 50.3% in 41 patients with HCC and PVTT treated with SBRT. In 2018, Rim et al. [9] further confirmed that no significant differences in the OS rate were observed after undergoing 3DCRT, selective internal radiation therapy (SIRT), and SBRT. Furthermore, SBRT had the highest response rate, followed by 3DCRT and SIRT. A retrospective study by Matsuo et al. [16] reported the efficacy of SBRT compared with that of 3DCRT in the treatment of PVTT, demonstrating that the 1-year OS rates were 49.3 and 29.3%, and the 1-year local progression rates were 20.4 and 43.6%, respectively. Based on the results of various studies, SBRT appears to be more therapeutically effective in patients with HCC and PVTT, especially in those who are ineligible for surgery or TACE.

There are only a few published reports on the efficacy of SBRT for PVTT; for SBRT, the response rates range from 44.4 to 75.6%, the median survival durations range from 8 to 13 months, and the 1-year OS rates range from 43.2 to 50.3% [14, 16, 17]. In the present study, the median survival duration, 1-year OS rate, and objective response rate (CR + PR) in patients with HCC and PVTT were 11.5 months, 47.5, and 79.17%, respectively, comparable to and consistent with those reported in previous works. A prospective study by Bujold et al. [28] reported a 1-year OS rate of 44% in 56 patients with HCC and tumour thrombosis, treated with SBRT; this demonstrated that our results are consistent with those of most previous studies. A recent study showed a median survival period of 10 months after performing SBRT and a median follow-up period of 31 months for patients with HCC and PVTT. The results were more convincing as the follow-up period was longer [29]. In our study, SBRT was well tolerated, and no treatment-related serious adverse events or deaths were observed. The low toxicity of SBRT was consistent with the results of other SBRT studies [14, 19].

In this study, the multivariate analyses revealed that the clinical T stage, age, presence of cirrhosis, AFP levels, and haemoglobin levels were significant independent predictors of OS. Three factors, namely, the presence of cirrhosis, AFP levels, and haemoglobin levels are related to underlying liver function, and clinical T stage is associated with the tumour status. Thus, this study also confirmed that the tumour status and liver function were closely related to the OS of patients with HCC and PVTT. In addition, the reliability of the BCLC classification has been confirmed. Prognostic factors for HCC have been shown to include tumour size, tumour type, tumour stage, presence of cirrhosis, Child-Pugh class, AFP level, and serological indicators of liver function [30–33], consistent with our study findings.

A distinctive feature of this study was the development of an individualised prediction model to predict the OS of each patient and to improve treatment recommendations for patients. Based on the final regression analysis, a nomogram was constructed involving the five most significant risk factors for predicting OS. Nomograms have proved useful in assessing the prognosis of many patients with cancer. Nomograms reflect the characteristics of the tumour and the state of the host, comprising additional clinical parameters. Thus, nomograms are considered more advantageous than the traditional staging methods. Some researchers have proposed its use as an alternative approach or even as a new standard to guide cancer treatment [34, 35].

In the present study, cases of HCC with PVTT were considered as advanced liver cancer cases, and patients had similar staging information, making the prediction of OS based on TNM stage, difficult. Moreover, the tumour size, lymph node status, and metastasis status in TNM staging are based on gross anatomical information, which may not be fully consistent. Many studies have demonstrated that a radiomics nomogram is superior to a clinical nomogram; this may be because a radiomics nomogram provides a non-invasive assessment reflecting intra-tumour heterogeneity. Therefore, the addition of a radiomics signature to our nomogram would be beneficial for increasing its prognostic value [36–38].

This study had several limitations. Especially, it was a retrospective study with a small sample size, which might have led to selection bias. The results of this study need to be further confirmed by a prospective, multicentre, randomised controlled trial. Clinical T stage, age, presence of cirrhosis, AFP levels, and haemoglobin levels were included in the nomogram as independent predictive factors. Because of the small number of indicators included in the study, more effective indicators may not have been included; thus, the prediction efficiency of the model requires further improvement. Another limitation of this study was the lack of validation based on independent data sets. Limited by the small sample size and the single-centre design, the survey cohort could not be divided into a training and a verification group.

## Conclusions

This study demonstrated that SBRT is an effective treatment for HCC and that clinical T stage, presence of cirrhosis, age, AFP levels, and haemoglobin levels were independent prognostic factors for OS. The clinical nomogram may be used for OS prediction in patients with HCC and PVTT who underwent SBRT.

## Data Availability

The datasets used and/or analysed during the current study are available from the corresponding author on reasonable request.

## Abbreviations

3DCRT: three-dimensional conformal radiotherapy
4DCT: four-dimensional computed tomography
AFP: alpha-fetoprotein
ALB: albumin
AUC: area under the receiver operating characteristic curve
BCLC: Barcelona Clinic Liver Cancer
CR: complete response
DCA: decision curve analysis
ECOG: Eastern Cooperative Oncology Group
GTV: gross tumour volume
HCC: hepatocellular carcinoma
HGB: haemoglobin
ITV: Internal target volume
IVCTT: inferior vena cava tumour thrombosis
MRI: magnetic resonance imaging
OS: overall survival
PR: partial response
PTV: planning target volume
PVTT: portal vein tumour thrombosis
SBRT: stereotactic body radiotherapy
SD: stable disease
SIRT: selective internal radiation therapy
TACE: transarterial chemoembolisation
RFA: radiofrequency ablation
